# The evolution of single-use duodenoscope utilization at a large-volume endoscopic retrograde cholangiopancreatography tertiary care center

**DOI:** 10.1016/j.igie.2025.03.014

**Published:** 2025-04-04

**Authors:** Yervant Ichkhanian, Hashem N. Albunni, Aditya Gutta, James L. Watkins, Evan L. Fogel, Jeffrey J. Easler, Nasir Saleem, Mark A. Gromski

**Affiliations:** 1 Division of Gastroenterology and Hepatology, Indiana University School of Medicine, Indianapolis, Indiana, USA; 2 Department of Internal Medicine, Indiana University School of Medicine, Indianapolis, Indiana, USA; 3 Virginia Mason Medical Center, Seattle, Washington

## Abstract

**Background and Aims:**

Previous studies have shown promising outcomes with single-use duodenoscopes, often examining only 1 version. The aim of this study was to evaluate the long-term outcomes across multiple models at a large referral center.

**Methods:**

Endoscopic retrograde cholangiopancreatography (ERCP) cases (July 2020-September 2023) were retrospectively reviewed to assess technical success, defined as successful cannulation without switching devices. A 3-tier system guided the use of single-use duodenoscopes: recommended for drug-resistant cases (Tier 1), considered for immunosuppressed patients (Tier 2), and discouraged in routine cases (Tier 3).

**Results:**

Of 8375 ERCPs, 267 (3.2%) involved single-use duodenoscopes (Tier 1, 25%; Tier 2, 54%; and Tier 3, 21%). The majority focused on the biliary system (91%), with 9% targeting the pancreatic duct. Technical success was achieved in 94%. Technical failures were primarily due to poor imaging (53%) and inadequate maneuverability (47%). Adverse events were reported in 3% of cases. A gradual increase was observed in ERCPs using single-use duodenoscopes, from 2.6% in 2020 to 4.7% in 2023, alongside a notable decrease in Tier 3 usage from 29% to 14%. The use for native papillae rose from 9.8% to 21%, while pancreatic duct interventions dropped significantly from 15% to 4.8%. Junior faculty performed 84% of procedures, reporting greater satisfaction than senior faculty. Improvements in scope stiffness, image stability, and elevator functionality were noted with newer iterations.

**Conclusions:**

This study offers real-world evidence of successful single-use duodenoscope integration into a high-volume practice, with growing use and increased endoscopist satisfaction over time.

Outbreaks linked to the use of contaminated duodenoscopes have been well documented.^1^, ^2^, ^3^, ^4^ Infections associated with these outbreaks pose a significantly heightened risk of clinical deterioration, especially when they involve multidrug-resistant organisms, particularly in immunocompromised individuals.^5^^,^^6^ Patient risks and directives from government authorities led to significant investment in the development of enhanced practices, device modifications, and novel devices to mitigate the risk of infections.

One advancement in duodenoscope design has been the introduction of disposable end caps, which are discarded after each use,^7^^,^^8^ allowing better access to the elevator mechanism during the reprocessing phase, facilitating more effective disinfection. However, complete eradication of bacterial growth remains challenging due to several factors,^9^^,^^10^ including human error in reprocessing practices, difficulties cleaning air and water channels, damage to the inner sheaths of working channels, and the formation of biofilms.^11^^,^^12^ Fully disposable, sterile single-use duodenoscopes have been developed, effectively eliminating the risk of bacterial transmission from contaminated devices.^6^^,^^13^^,^^14^

Although earlier studies assessed the utilization and performance of single-use duodenoscopes, they typically focused on short-term use, often involving a single iteration of the device.^1^, ^2^, ^3^, ^4^ The current study represents the first comprehensive examination of the usage and performance of single-use duodenoscopes since the U.S. Food and Drug Administration clearance up to the present. This allowed for an in-depth analysis of practice patterns, performance trends, and the impact of user experience over multiple duodenoscope iterations. Data on the real-life, long-term integration of single-use duodenoscopes into clinical practice, including utility, outcomes, and endoscopist satisfaction scores, have not been previously reported. Accordingly, we aim to present our experiences with the integration of single-use duodenoscopes into our practice.

## Methods

This study was a retrospective review of a prospectively collected endoscope assessment database. Data were prospectively collected for each procedure utilizing a single-use duodenoscope and entered into a database. The cohort of patients underwent endoscopic retrograde cholangio-pancreatography (ERCP) using single-use duodenoscopes at our center between July 2020 and September 2023. The study received approval from the Institutional Review Board of the Indiana University School of Medicine. The criteria for utilizing single-use duodenoscopes were developed based on a 3-tier adoption recommendation system established by our institution:

• Tier 1: high risk for harboring drug-resistant organisms within the biliary tract; recommend use of single-use duodenoscope.
• Tier 2: patients with increased risk of clinical decompensation due to immunosuppressed state; consider use of single-use duodenoscope.
• Tier 3, all other routine cases; discourage the routine use of single-use duodenoscope.^1^

### Procedures

All procedures were performed by gastroenterologists with dedicated training in therapeutic endoscopy at Indiana University School of Medicine University Hospital, a high-volume tertiary care center averaging 2700 to 2900 ERCPs annually during the study period. The single-use duodenoscopes used included the EXALT Model D single-use duodenoscope (Boston Scientific, Marlborough, Mass, USA) and the Ambu aScope Duodeno (Ambu, Copenhagen, Denmark). Throughout the study period, 3 different iterations of the EXALT Model D single-use duodenoscope were used: the first iteration was utilized from July 2020 to February 2022, the second from February 2022 to May 2023, and the third was introduced in May 2023. All the procedures were performed with the patient under general anesthesia, and standard-of-care ERCP techniques were used.

### Definitions and outcomes

A prospectively maintained database using REDCap (Research Electronic Data Capture) was used for this study. The complexity of the ERCP procedures and the severity of adverse events (intraprocedural and postprocedural) were reported based on the American Society for Gastrointestinal Endoscopy (ASGE) grading system for ERCP complexity^15^ and ASGE’s lexicon for severity grading system,^16^ respectively. Post-ERCP adverse events were assessed by reviewing our institution’s records as well as outside hospital admissions and emergency department visits via shared hospital records.

The primary outcome measure was technical success, defined as the successful cannulation and the completion of ERCP procedures for the intended indication without the need to switch to a reusable duodenoscope or to use a different single-use duodenoscope. Secondary outcomes included endoscopist-reported satisfaction, which was assessed by using a questionnaire formatted on a 5-point Likert scale, where 1 indicates “completely fails expectations” and 5 represents “definitely exceeds expectations.” The endoscopists were instructed to compare the single-use duodenoscopes with the TJF-160/180 model duodenoscope (Olympus, Tokyo, Japan), which was the model of duodenoscopes in primary use at the time of creation of the assessment tool. This tool facilitated a standardized assessment of the procedural performance of individual single-use duodenoscopes. In cases in which a fellow participated, they contributed to the completion of the assessment questionnaire. Additional secondary outcomes included the rate and severity of adverse events, as well as the success rate of cannulating both native and non-native papillae.

Subgroup analyses were conducted to evaluate utility, outcomes, and endoscopist-reported satisfaction scores across various single-use duodenoscope manufacturers and iterations, as well as between junior and senior faculty. Endoscopists were classified as junior if they were within 10 years of completing their therapeutic endoscopy training and as senior if they were >10 years from their therapeutic endoscopy training.

### Statistical analysis

De-identified data were documented by using a pre-designed spreadsheet. Descriptive statistics were analyzed with SPSS version 29.0 (IBM SPSS Statistics, IBM Corporation, Armonk, NY, USA). Continuous variables that were normally distributed are presented as means (standard deviation), and those with skewed distributions are reported as medians (interquartile range [IQR]). Categorical data were summarized by using counts and percentages. To compare outcomes, various statistical tests were used, including the Student t test, the Mann-Whitney *U* test, and the Fisher exact test.

## Results

**Figure 1:**
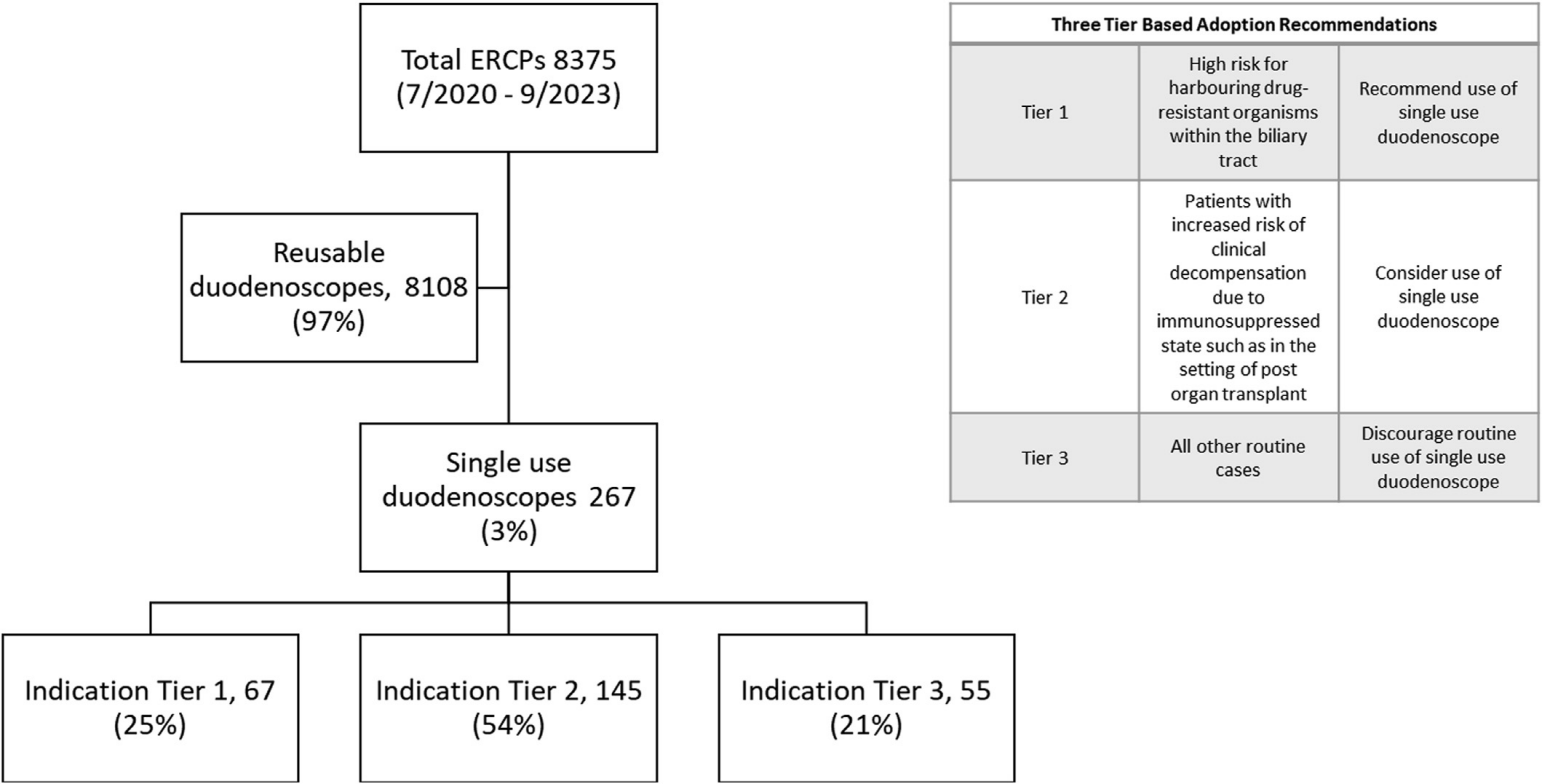
An algorithm illustrating the enrolled patients and indications for the use of single-use duodenoscopes. *ERCP*, endoscopic retrograde cholangiopancreatography.

During the study period, a total of 8375 ERCPs were performed at our institution between July 2020 and September 2023; of these, 267 (3.2%) were performed with single-use duodenoscopes in 251 patients (mean age, 56.8 (16) years; 56% female). The utilization of single-use duodenoscopes was based on clinical indications: Tier 1 indications were noted in 67 patients (25%), Tier 2 in 145 patients (54%), and Tier 3 in 55 patients (21%) (Fig. 1). These procedures were performed by a total of 8 endoscopists, comprising 5 senior faculty members and 3 junior faculty members. Training fellows were involved in 32 (12%) cases.

Among the patients who underwent ERCP using single-use duodenoscopes, 48 (18%) had a native papilla at the time of the initial ERCP, indicating no prior sphincterotomy and cannulation. The majority of the targeted interventions were biliary in nature, accounting for 243 cases (91%), whereas pancreatic duct (PD) interventions comprised 24 cases (9%). The primary indications for biliary interventions were as follows: intrahepatic hilar strictures in 63 patients (24%), extrahepatic biliary strictures in 60 patients (22%), anastomotic strictures in 57 patients (21%), choledocholithiasis in 39 patients (15%), primary sclerosing cholangitis or COVID cholangiopathy in 35 patients (13%), biliary leaks in 7 patients (2.6%), diagnostic cholangioscopy in 2 patients (.7%), cystic duct stone removal in 2 patients (.7%), and sphincter of Oddi dysfunction in 2 patients (.7%). For pancreatic interventions, the most common indications included PD leaks in 2 patients (8.3%), stones in 5 patients (21%), strictures in 6 patients (25%), stent removal in 8 patients (33%), and stent exchange in 2 patients (8.3%).

### Procedure difficulty

Overall, the complexity of the performed ERCPs was classified as ASGE grade 3 in 127 patients (48%), followed by grade 2 in 86 patients (32%), and grade 1 in 54 patients (20%) (Table 1). The median number of attempts required to successfully cannulate the target duct was 1 (IQR, 1-2). The median time from the mouth to the papilla was 1 minute (IQR, 1-2 minutes), and the median time for cannulation was 2 minutes (IQR, 1-5 minutes).

**Table 1:** Outcomes based on the ERCP complexity

Outcome	ASGE complexity			*P* value
	Grade 1	Grade 2	Grade 3	
No. of patients	54 (20)	86 (32)	127 (48)	
Fellows involved	9 (17)	13 (15)	10 (7.9)	.12
Median procedure time, min	16 (13-26)	21.5 (15-39.3)	29 (21-44)	.08
Crossover to reusable	2 (3.7)	4 (4.7)	9 (7)	.06
Native papilla	3 (5.6)	27 (31)	18 (14)	.34
Cannulation attempts, number	1 (1-3)	2 (1-6)	1 (1-4)	.18
Mouth to papilla time, number	1 (1-2)	1 (1-2)	1 (1-2)	.6
Cannulation time, number	1 (1-2)	2 (1-3)	1 (1-2)	.4

Values are n (%) or median (IQR).

*ASGE*, American Society for Gastrointestinal Endoscopy; *ERCP*, endoscopic retrograde cholangiopancreatography.

Of the 48 patients with native papilla at the time of the ERCP, advanced cannulation techniques were used in 6 patients (12.5%), which included placing PD stents in 4 (67%) patients and using the precut sphincterotomy technique in 2 (33%) patients. Biliary endoscopic sphincterotomy was performed in 49 patients (18%) and endoscopic pancreatic sphincterotomy in 7 patients (2.6%).

### Procedure outcomes

The average total procedure time was 29.1 (18) minutes, with technical success achieved in 252 cases (94%). Among the 15 patients who experienced technical failures requiring a switch to a reusable duodenoscope, 7 patients (46%) were reported to have a native papilla, with the majority of targeted interventions being biliary in nature, accounting for 12 cases (80%). The complexity of the ERCP procedures performed was predominantly ASGE grade 3 in 9 patients (60%), followed by grade 2 in 4 patients (27%) and grade 1 in 2 patients (15%) (*P* = .04). The most frequently cited reasons for the failed ERCPs (multiple reasons could be identified for each case) were poor imaging in 8 patients (53%), inadequate maneuverability in 7 patients (47%), suboptimal elevator function in 3 patients (20%), poor insufflation in 1 patient (6.7%), and failure to advance or perform cholangioscopy in 1 patient (6.7%) (Table 2). No additional technical failures were reported after transitioning to reusable duodenoscopes among patients who initially experienced technical failures with single-use duodenoscopes.

**Table 2:** Case details on technical failure cases

Reason for switch over to reusable duodenoscope	No. of patients	Type of duodenoscope	The iteration of the duodenoscope	Single-use duodenoscope indication tier	Indication	ASGE ERCP complexity	Native papilla	Target duct
Suboptimal elevator function	1	EXALT	First	2	Post liver transplant biliary stricture	1	Yes	Biliary
Poor imaging	4	EXALT	First	1	Extrahepatic biliary stricture	2	No	Biliary
		EXALT	Second	1	Portal biliopathy	2	No	Biliary
		EXALT	Second	3	Hilar stricture	3	No	Biliary
		EXALT	Third	3	Pancreatic duct stricture	3	Yes	Pancreatic
Inadequate maneuverability	5	EXALT	First	3	PD stricture	3	No	Pancreatic
		EXALT	First	3	PD stone	3	No	Pancreatic
		Ambu	–	3	Choledocholithiasis	2	Yes	Biliary
		EXALT	Second	2	Bile leak	1	Yes	Biliary
		EXALT	Third	2	Hilar stricture	3	Yes	Biliary
Poor insufflation + poor imaging	1	EXALT	First	2	Cystic duct stone removal	3	No	Biliary
Inadequate maneuverability + poor imaging	2	EXALT	First	3	Choledocholithiasis	2	Yes	Biliary
		EXALT	First		Choledocholithiasis	3	Yes	Biliary
Suboptimal elevator function + failure to advance/perform cholangioscopy	1	Ambu	–	2	Posttransplant anastomotic stricture	3	No	Biliary
Poor imaging + Suboptimal elevator function	1	EXALT	Third	2	Extrahepatic biliary stricture	3	No	Biliary

*ASGE*, American Society for Gastrointestinal Endoscopy; *ERCP*, endoscopic retrograde cholangiopancreatography; *PD*, pancreatic duct.

During the study period, there were a total of 8 adverse events (3%). Two patients experienced bleeding: 1 due to scope trauma involving a Schatzki ring at the gastroesophageal junction that was managed by endoscopic band ligation, and the other due to suspected bleeding from a choledochal varices that was managed by the placement of a covered metallic biliary stent. In addition, 5 patients reported mild to moderate abdominal pain postprocedure, all with normal lipase levels; they were treated with supportive care, and no diagnosis of post-ERCP pancreatitis was made. Finally, 1 patient with underlying primary sclerosing cholangitis developed *Serratia* bacteremia 2 days after the ERCP, classified as moderate, and was treated with antibiotics (Table 3).

**Table 3:** Case details of AEs

AE	Total no. of AEs	Details	ASGE lexicon severity scoring for AE	ASGE ERCP complexity scoring grade	Management	Outcome
Bleeding	2	Trauma involving a Schatzki ring at the gastroesophageal junction Suspected bleeding from a choledochal varices	Mild	3	Intraprocedurally, 3 bands were successfully placed in the lower third of the esophagus Intraprocedurally, stent placement (covered metallic biliary stent) required to achieve hemostasias	Successful control of the bleeding
Postprocedure pain	5	Non-pancreatitis–type abdominal pain	Mild	3, 2	Supportive	Successfully controlled symptoms
Immediate infection	1	2 days after ERCP	Moderate	3	Antibiotics	Successfully treated without AEs

*AEs*, Adverse events; *ASGE*, American Society for Gastrointestinal Endoscopy; *ERCP*, endoscopic retrograde cholangiopancreatography.

### Trends in the use of single-use duodenoscopes

**Figure 2:**
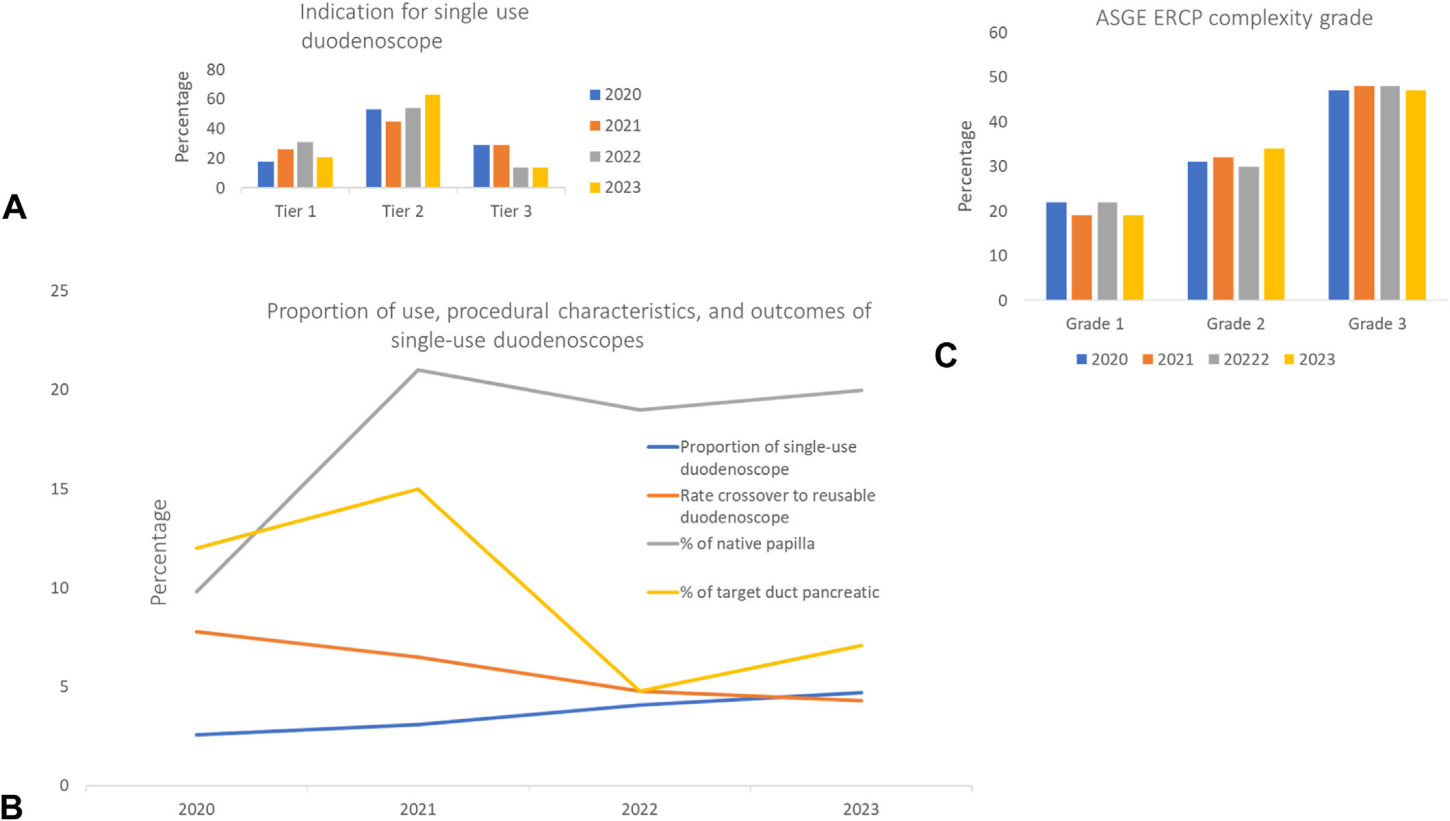
Summary of the trends regarding use of single-use duodenoscopes over time. **A,** Indications for single-use duodenoscope use based on the 3-tier categorization over the course of the study. **B,** Trends in the proportion of use, procedural characteristics, and outcomes of single-use duodenoscopes over time. **C,** Trends in single-use duodenoscope utilization over time, stratified according to endoscopic retrograde cholangiopancreatography (ERCP) procedural complexity as per the American Society for Gastrointestinal Endoscopy (ASGE) complexity grade.

Over the >3-year duration of the study, there was an increase in the percentage of ERCP procedures performed with single-use duodenoscopes: 51 cases (2.6%) in 2020, 62 cases (3.1%) in 2021, 83 cases (4.1%) in 2022, and 70 cases (4.7%) in 2023, showing a statistically significant increase from 2020 to 2023 (*P* = .02). In addition, there was a notable reduction in the number of patients undergoing ERCP with single-use duodenoscopes for Tier 3 indications, decreasing from 15 cases (29%) in 2020 to 10 cases (14%) in 2023 (*P* = .021) (Fig. 2, Table 4).

**Table 4:** Trends in the use of single-use duodenoscopes

Year	No. of procedures	Fellow involved	Type of duodenoscope	Procedure time (min)	Single-use duodenoscope indication: Tier 1, Tier 2, Tier 3	ASGE ERCP complexity: Tier 1, Tier 2, Tier 3	Native papilla	PD targeted therapies	Cannulation attempts	Mouth to papilla time (min)	Cannulation time (min)	Crossover to reusable
2020	51 (2.6)	0	EXALT, 51 (100)	28 (13-47)	9 (18), 27 (53), 15 (29)	11 (22), 16 (31), 24 (47)	5 (9.8)	6 (12)	1 (1-2.5)	1 (1-2)	1 (1-2.25)	4 (7.8)
2021	62 (3.1)	4 (6.5)	EXALT, 47 (76) Ambu 15 (24)	26 (17-42.5)	16 (26), 28 (45), 18 (29)	12 (19), 20 (32), 30 (48)	12 (19)	9 (15)	3 (1-5.5)	1 (1-2)	2 (1-3)	4 (6.5)
2022	83 (4.1)	5 (6)	EXALT, 72 (87); Ambu, 11 (13)	25.5 (17.75-32.3)	26 (31), 45 (54), 12 (14)	18 (22), 25 (30), 40 (48)	16 (19)	4 (4.8)	1 (1-2)	1 (1-2)	1 (1-4)	4 (4.8)
2023	71 (4.7)	10 (14)	EXALT, 71 (100)	21 (15-32)	16 (23), 45 (63), 10 (14)	13 (18), 25 (35), 33 (46)	15 (21)	5 (7.1)	1 (1-2)	1 (1-1.25)	1 (1-4)	3 (4.3)

Values are n (%) or median (interquartile range).

*ASGE*, American Society for Gastrointestinal Endoscopy; *ERCP*, endoscopic retrograde cholangiopancreatography; *PD*, pancreatic duct.

During the study, the use of single-use duodenoscopes for patients with native papillae increased significantly from 5 cases (9.8%) in 2020 to 13 cases (21%) in 2021 (*P* = .031). However, beginning in 2021, there was a decline in their use for interventions targeting the PD, with a significant decrease from 9 cases (15%) in 2021 to 4 cases (4.8%) in 2022 (*P* = .042). Overall, there was no significant change in the complexity of ERCP procedures performed with single-use duodenoscopes over time. In addition, there was a nonsignificant downward trend in technical failure rates, decreasing from 4 cases (7.8%) in 2020 to 3 cases (4.3%) in 2023 (*P* = .062) (Fig. 1, Table 4).

### Endoscopists’ satisfaction

**Figure 3:**
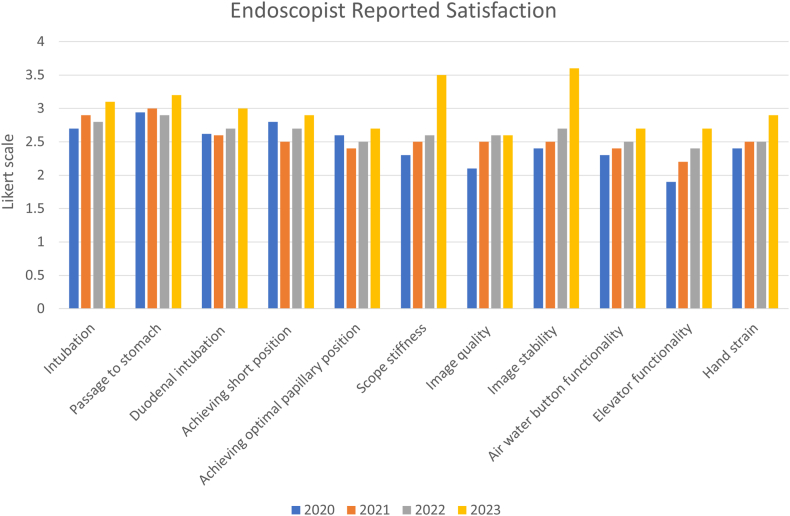
Endoscopist-reported satisfaction score on the single-use duodenoscopes over time.

The mean overall satisfaction scores reported were 2.4 (0.2) in 2020, 2.2 (0.3) in 2021, 2.3 (0.1) in 2022, and 2.6 (0.3) in 2023, indicating a statistically significant improvement from 2021 to 2023 (*P* = .047). A score of 3 was “consistent with performance of Olympus TJF 160/180 model.” The lowest satisfaction rating was associated with the functionality of the elevator mechanism, which received a score of 1.89. Two parameters showed significant improvement from 2022 to 2023: "scope stiffness" increased from 2.57 (0.1) to 3.5 (0.3) (*P* = .031), and "image stability" improved from 2.61 (0.2) to 3.62 (0.4) (*P* = .021) (Fig. 3). Although there was no significant difference in overall satisfaction scores among the 3 ASGE grades of ERCP complexity, the parameter of “achieving optimal papillary position” was notably lower in ERCPs categorized as ASGE complexity grade 3, with a score of 1.89 (0.42), compared with grade 2 and grade 1 ERCPs, which had scores of 2.43 (0.23) (*P* = .012).

### Subgroup analyses based on the endoscopists’ level of expertise

The majority of ERCP procedures performed using single-use duodenoscopes were conducted by junior faculty, accounting for 223 cases (84%); senior faculty performed 44 cases (16%). In 2020, senior faculty performed 28 cases (55%) of the ERCPs with a single-use duodenoscope. This number decreased to 8 cases (13%) in 2021 and 1 case (1.2%) in 2022, and then increased slightly to 7 cases (10%) in 2023. Conversely, in 2020, junior faculty performed 23 cases (45%) of the ERCPs with a single-use duodenoscope, and this increased to 54 cases (87%) in 2021, 82 cases (98.8%) in 2022, and 64 cases (90%) in 2023. Junior faculty were more likely to use single-use duodenoscopes for Tier 1 indications, with 62 cases (28%), compared with 5 cases (11%) among senior faculty (*P* = .011). However, they were less likely to perform ERCPs targeting the PD, with 18 cases (8%) versus 6 cases (14%) for senior faculty (*P* = .041). A greater number of ERCPs classified as ASGE complexity grade 3 were performed by junior faculty, with 105 cases (47%) compared with 22 cases (5%) by senior faculty (*P* = .04). Among the total of 15 patients with technical failures, 11 (73%) were procedures performed by junior faculty, while 4 (27%) were conducted by senior faculty (*P* = .21).

Overall satisfaction scores improved from 2020 to 2023 for both junior and senior faculty. However, only junior faculty exhibited a statistically significant increase in their average satisfaction score, from 2.2 (0.5) to 2.7 (0.4) between 2021 and 2023 (*P* = .032). The parameter “scope stiffness” had a significantly lower average score among senior faculty at 2.1 (0.3) compared with junior faculty at 3.1 (0.5) (*P* = .022).

### Subgroup analysis based on the different manufacturer and iterations of the single-use duodenoscopes

Of the single-use duodenoscopes used, 241 (90%) were manufactured by Boston Scientific, specifically the EXALT Model D, while the remaining 26 (10%) were produced by Ambu, known as the aScope Duodeno. There were no significant differences in terms of utility, complexity, rates of crossover, and outcomes between the different manufacturers.

**Figure 4:**
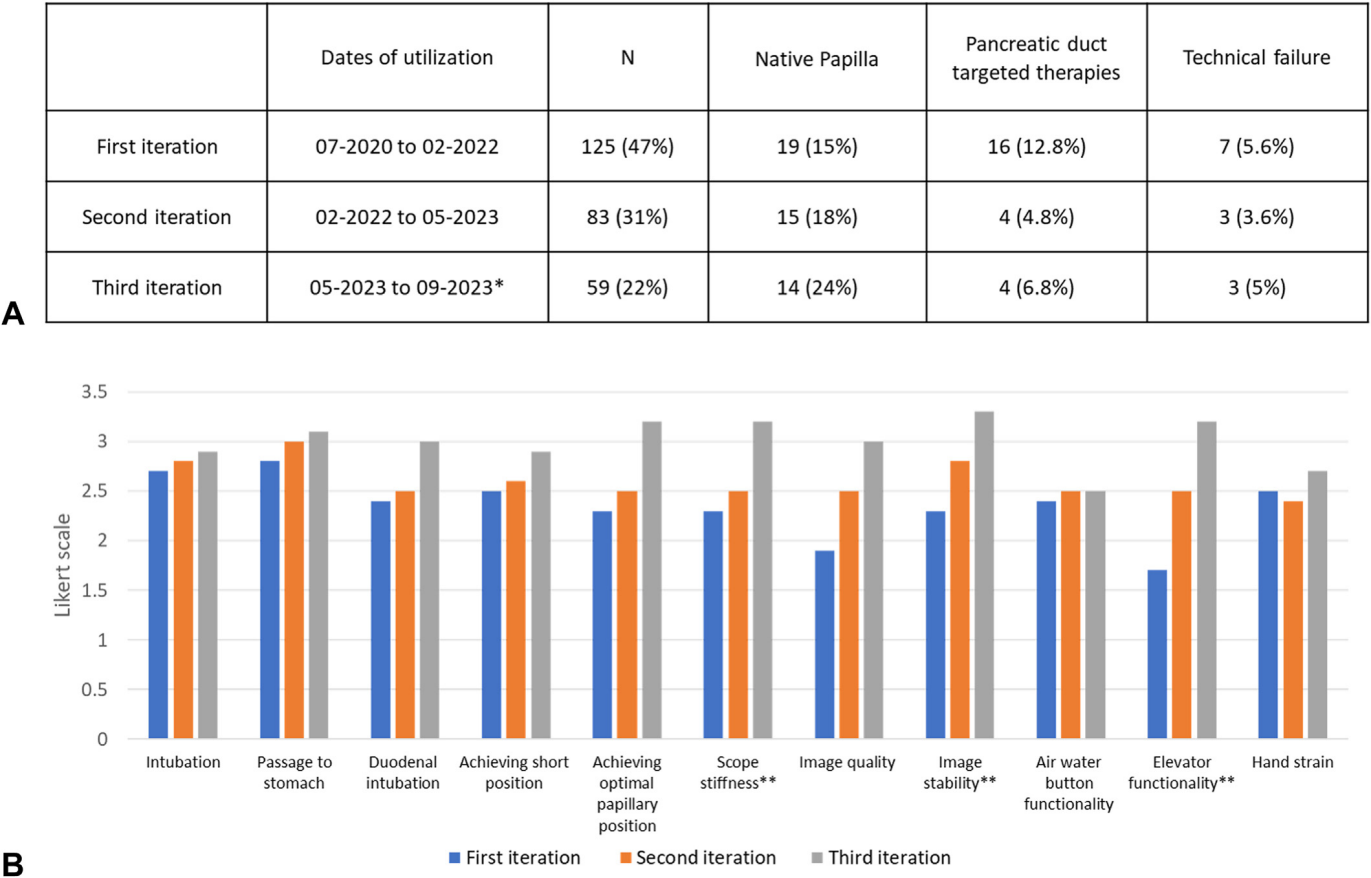
Procedural details, outcomes, and the endoscopist-reported satisfaction per the EXALT Model D single-use duodenoscope iteration. **A,** Procedural characteristics and outcomes stratified according to the iteration of the single-use duodenoscope. **B,** Endoscopist-reported satisfaction by the iteration of the single-use duodenoscope. ∗End of the study duration. ∗∗Endoscopist-reported satisfaction parameters that significantly improved from the first to the third version.

During the study, 3 different versions of the EXALT Model D single-use duodenoscope were used. Nearly one half of the ERCP procedures were conducted using the first version of the EXALT Model D, accounting for 125 cases (47%), followed by the second version with 83 cases (31%), and the third version with 59 cases (22%) (Fig. 4). The ERCPs performed with the third version had the highest percentage of native papillae, at 14 cases (24%), whereas the first version had the highest rate of procedures targeting the PD, with 16 cases (12.8%). The rates of technical failure among the 3 versions were 7 cases (5.6%) for the first version, 3 cases (3.6%) for the second version, and 3 cases (5%) for the third version (*P* = .24). Overall, there was an improvement in all parameters of endoscopist-reported satisfaction scores, with significant enhancements noted in scope stiffness, image stability, and elevator functionality from the first to the third version, with *P* values of .032, .041, and .02, respectively.

## Discussion

Duodenoscope-related infections have garnered significant attention related to the problem and management of risk for patients undergoing ERCP. To mitigate this risk, single-use duodenoscopes have been introduced as a sterile alternative, yielding promising initial outcomes.^1^^,^^6^^,^^14^ However, as with any technological innovation in endoscopy, the adoption of single-use duodenoscopes tends to occur gradually, influenced by institution-specific satisfaction rates, endoscopist preferences, and the specific clinical scenarios in which the device is utilized. In this study, we documented our clinical experience with incorporating single-use duodenoscopes into our high-volume ERCP practice. A total of 267 patients underwent ERCP using single-use duodenoscopes over a 38-month period, translating to 1 single-use ERCP for every 69 procedures conducted with reusable duodenoscopes. To our knowledge, this represents the largest single-center cohort of patients who underwent ERCP using single-use duodenoscopes.

Our institution implemented a 3-tier adoption recommendation system, suggesting that patients in Tier 1 (those at high risk of harboring drug-resistant organisms) should be prioritized for single-use duodenoscope use, whereas Tier 2 (immunocompromised patients) indicates a preference for these devices. Notably, the application of these recommendations was left to the discretion of the endoscopists, resulting in 21% of cases categorized as Tier 3, in which routine use of single-use duodenoscopes is not typically advised. This shows that our internal guidance documents are not a command for our ERCP doctors but that the ERCP doctor may use their judgment to make the best decision on equipment to achieve their clinical goals. During the study period, however, there was a decline in the percentage of Tier 3 patients undergoing ERCP with single-use duodenoscopes, decreasing from 29% in 2020 to 14% in 2023, which indicated the overall trend in minimizing the overutilization of single-use duodenoscopes for cases. This trend may suggest that endoscopists initially used single-use duodenoscopes for more routine cases to become familiar and comfortable with the technology, before expanding their use to higher-risk patient populations in Tiers 1 and 2.

Throughout the study period, there was no significant change in the complexity of ERCPs performed using single-use duodenoscopes. However, there was a notable increase in the utilization of single-use duodenoscopes for patients with native papillae, rising from 9.8% in 2020 to 21% in 2023. In contrast, there was a significant reduction in their use for PD interventions, decreasing from 15% in 2021 to 4.8% in 2022. This trend may suggest that endoscopists became increasingly comfortable with using single-use duodenoscopes for cannulating native papillae but began to reduce their application for PD interventions, likely due to a relative low likelihood that a pancreatic intervention would fit into the Tier 1 or 2 recommendation for consideration for usage.

Technical failures that required switching to a reusable duodenoscope occurred in 15 patients (6%), a finding consistent with previously reported literature.^6^ The reasons for these technical failures were exclusively related to mechanical malfunctions or suboptimal performance of the duodenoscope, with “poor visualization” being the predominant issue in 53% of cases and “inadequate maneuverability” in 47%. Notably, throughout the study, “scope stiffness” and “image stability” emerged as parameters that showed significant improvement with time. In terms of safety, the overall rate of adverse events was low, at only 3%, with no reports of post-ERCP pancreatitis. This rate aligns with what has been documented in existing literature regarding the safety of ERCP procedures in general and the use of single-use duodenoscopes.^17^ A lower-than-expected incidence of post-ERCP pancreatitis might also be attributed to the fact that only 18% of the patients had native papillae.

It is recognized that endoscopists at various stages of their careers may exhibit differing levels of receptiveness to technological innovations, as well as diverging satisfaction trends.^18^^,^^19^ In our study, the single-use duodenoscope was predominantly utilized by junior faculty, accounting for 84% of its use. This trend indicates a more conservative adoption approach among senior faculty regarding the performance of ERCPs with single-use duodenoscopes. For example, senior faculty conducted fewer ERCPs classified as ASGE complexity grade 3, with only 5% compared with 47% performed by junior faculty. In addition, junior faculty utilized single-use duodenoscopes more frequently for Tier 1 indications, with 28% usage versus 11% among senior faculty. In addition, the decrease in the total number of ERCPs performed by senior faculty from 2020 to 2022 may indicate that initial exposure for the senior faculty were to the very earliest versions of the single-use duodenoscopes. In this study, senior faculty utilized more older iterations of the duodenoscopes, which, based on our user data, had significant disadvantages in terms of “scope stiffness,” “image stability,” and “elevator functionality” compared with the newer models. This early exposure may have limited willingness to use the scope for future cases.

This study has several limitations, including its design as a retrospective review of a prospectively collected database and the potential for selection bias. A significant limitation also pertains to pandemic-related restrictions, which may have affected the volume, timing, and utilization of the novel device for ERCP procedures. Nevertheless, to our knowledge, this analysis represents the largest single-center cohort examining single-use duodenoscopes in a diverse patient population, thus minimizing the heterogeneity and biases often associated with multicenter studies. Other aspects of utilization of single-use duodenoscopes include cost considerations and environmental impact of use. Further study into the aforementioned factors, including long-term assessment of the incidence of duodenoscope-related infection, will be important to steer the discourse on utilization of novel technology as well.

In conclusion, the current study provides a practical demonstration of the integration of a novel device within a high-volume clinical setting. Overall, the safety and efficacy of single-use duodenoscopes in our practice have been satisfactory. However, the adoption rate of single-use duodenoscopes among senior faculty remains low. It is noteworthy that various aspects of user experience have improved over time with the newer iterations of the single-use duodenoscope.

## Disclosure

The following author disclosed financial relationships: M.A. Gromski: consultant for 10.13039/100008497Boston Scientific and Ambu; and research support from 10.13039/100024146Olympus America and 10.13039/100010479Cook Medical. All other authors disclosed no financial relationships.
